# SUMO, a small, but powerful, regulator of double-strand break repair

**DOI:** 10.1098/rstb.2016.0281

**Published:** 2017-08-28

**Authors:** Alexander J. Garvin, Joanna R. Morris

**Affiliations:** Birmingham Centre for Genome Biology and Institute of Cancer and Genomic Sciences, Medical and Dental School, University of Birmingham, Edgbaston, Birmingham B15 2TT, UK

**Keywords:** SUMO, ubiquitin, double-strand break repair, SUMO protease

## Abstract

The response to a DNA double-stranded break in mammalian cells is a process of sensing and signalling the lesion. It results in halting the cell cycle and local transcription and in the mediation of the DNA repair process itself. The response is launched through a series of post-translational modification signalling events coordinated by phosphorylation and ubiquitination. More recently modifications of proteins by *S*mall *U*biquitin-like *MO*difier (SUMO) isoforms have also been found to be key to coordination of the response (Morris *et al.* 2009 *Nature* **462**, 886–890 (doi:10.1038/nature08593); Galanty *et al.* 2009 *Nature* **462**, 935–939 (doi:10.1038/nature08657)). However our understanding of the role of SUMOylation is slight compared with our growing knowledge of how ubiquitin drives signal amplification and key chromatin interactions. In this review we consider our current knowledge of how SUMO isoforms, SUMO conjugation machinery, SUMO proteases and SUMO-interacting proteins contribute to directing altered chromatin states and to repair-protein kinetics at a double-stranded DNA lesion in mammalian cells. We also consider the gaps in our understanding.

This article is part of the themed issue ‘Chromatin modifiers and remodellers in DNA repair and signalling’.

## 1. Overview of the SUMO system

**Figure 1.**
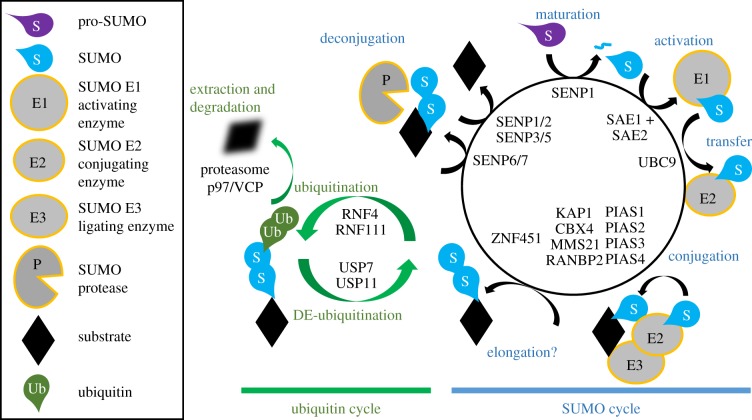
The SUMO cycle. SUMO1/2/3 isoforms are processed from their immature (pro)-forms into mature SUMO exposing the C-terminal GG motif. SENP1 is proposed to be the dominant SUMO maturing protease, although other SENPs have this ability *in vitro* [2]. Heterodimeric SUMO E1 enzyme adenylates the C-terminal diglycine followed by thioester formation with a Cys residue within the SAE2 subunit of the SUMO E1. The thioester is then transferred to a Cys residue within the E2 enzyme UBC9. SUMO can be conjugated directly to a Lys residue or residues on target proteins through the E2, or with the aid of SUMO E3 ligase. The E3 improves conjugation by either recruiting E2∼SUMO to a substrate or enhancing SUMO discharge from the E2 to the substrate. It is not yet clear if SUMO polymers are formed sequentially or if specialized E3 elongases (E4 enzymes) such as ZNF451 extend existing SUMO monomers [3,4]. SUMO polymers recruit multi-SIM-containing ubiquitin E3 ligases such as RNF4 and RNF111 to promote ubiquitination of the SUMO. This ubiquitinated SUMO can target the substrate for proteasome degradation. At least two de-ubiquitinating proteins, USP11 and USP7, are able to remove ubiquitin from SUMO polymers. SUMO polymers are disassembled via SENP6 and SENP7, while monomeric SUMO is deconjugated by SENPs1/2/3/5. Free SUMO released from substrates is then available to feed back into the conjugation cycle.

The *S*mall *U*biquitin-like *MO*difier (SUMO) system has relatively few enzymatic components, but unlike the ubiquitin system, there are several modifier isoforms (in mammals: SUMO1–5). SUMO proteins are approximately 12 kDa in size and have similarities to the three-dimensional structure of ubiquitin; however they share less than 20% amino acid identity and carry a different surface charge distribution [1]. Mature SUMO2 and SUMO3 are near identical (referred to as SUMO2/3) and share 50% identity with SUMO1. All SUMO proteins are expressed as an immature pro-form which must be cleaved prior to conjugation (figure 1). Whether SUMO4 is processed to a mature form [5–7] and whether the recently described SUMO5 is expressed [8] or a pseudogene [9] remains controversial. SUMO isoforms are conjugated to targets through three enzymatic steps: activation, involving the heterodimer E1 enzyme (SAE1 + SAE2); conjugation, involving the E2 enzyme (UBE2I/UBC9); and substrate modification, through the cooperation of the E2 with E3 protein ligases (figure 1). PIAS type SUMO E3 enzymes, which possess an SP-RING motif similar to the RING domain of many E3 ubiquitin ligases, are the best understood members.

**Figure 2.**
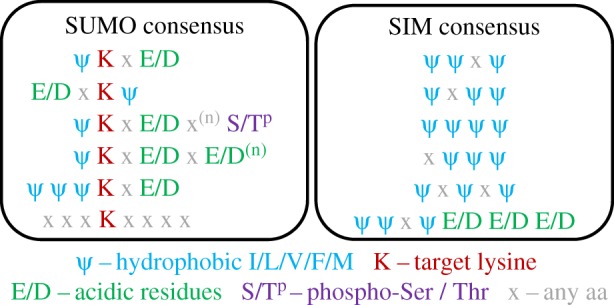
SUMO and SIM consensus (adapted from [10]). SUMO conjugation sites (left) are often found within the consensus ψKxE/D where ψ is a hydrophobic amino acid and x is any amino acid. Several variants including an inverted variant, and variants with additional acidic patches of amino acids or phosphorylated Ser/Thr residues have been identified. Approximately 25% of SUMO sites do not conform to any consensus [11]. Hydrophobic *S*UMO *I*nteracting *M*otifs (SIMs) (right).

SUMOylation is most frequent at a lysine within ψKxE/D-type motifs (figure 2) (where ψ represents a small hydrophobic amino acid and x any amino acid) which are directly recognized by the SUMO E2 UBC9 [12]. SUMOylation can be in the form of conjugation of a single SUMO to an acceptor lysine, or as in the ubiquitin system in the form of chains as polySUMO. These chains are generated on SUMO2/3 which, unlike SUMO1, possesses a ψKxE-type motif. SUMOylation can be affected by local environment; many SUMO consensus sites are embedded within patches of negatively charged amino acids [13]. Additionally, alterations in local charge, induced by phosphorylation of Ser/Thr residues, provide further control over target SUMOylation [14–16]. Under stress conditions SUMOylation frequently occurs at non-consensus lysines on target proteins [11].

In contrast to the array of proteases in the ubiquitin system just nine proteins have been identified with ability to process SUMO conjugates. In mammalian cells the two main classes of sentrin (SUMO)-specific proteases are related to the yeast Ulp1 (SENP1-3 and SENP5) and Ulp2 (SENP6 and 7) SUMO proteases. These enzymes differ in their maturation (C-terminal hydrolase) versus isopeptide cleavage activity and also in their preferences for different SUMO paralogues. Divergent amino termini of SENP proteins also result in differing sub-cellular localizations thereby restricting substrate access (reviewed in [2]). In addition a further class of proteases, named DeSI-1/2 (*D*e*S*UMOylated *I*sopeptidase 1/2) [17], with isopeptidase but little maturation capacity [18], and the previously assigned ubiquitin-specific protease (USPL1), which locates to Cajal bodies, have been revealed as SUMO isopeptidases [19]. Dramatic increases in SUMOylation profiles are reproducibly observed when SENP enzymes are blocked. For example, large increases in protein SUMOylation are observed in cells expressing mutants of SUMO that are resistant to SENP interaction [20], while heat shock inactivates SENP catalytic domains [21] and results in global increases in protein SUMOylation [22]. Thus SUMO proteases seem constitutively active under resting conditions and SUMO homeostasis appears to favour deSUMOylation.

SUMO can act as a docking signal promoting protein–protein interactions (reviewed in [23]). Various iterations of short hydrophobic patches known as SIMs (*S*UMO-*I*nteracting *M*otifs) have been identified with a common consensus of [ψ]-[ψ]-[x]-[ψ] where ψ most commonly represents isoleucine, leucine or valine [10,24–26] (figure 2). This peptide interacts with a groove on SUMO isoforms formed between the β2 strand and the first α-helix (reviewed in [27]). Two additional domains have been shown to interact with SUMO. The MYM class of zinc finger found in ZMYM3 and ZMYM2 interacts with the same surface patch on SUMO as SIM motifs [28]. The MYM fingers of ZMYM2 are important for interaction with HDAC1 within the LDS1/Co/REST/HDAC1 complex and are known to interact with multi-SUMOylated proteins [29,30]. Secondly the ZZ domain, found in HERC2 and acetyltransferase CBP, has also been shown to interact with SUMO1, but using a different surface from SIM motifs [31,32]. Thus multiple domains could interact with single SUMO moieties. It seems likely that additional SUMO binding domains remain to be identified.

Finally molecular mimicry of SUMO in the structurally related SUMO-like domains (SLDs) has the ability to disrupt and co-opt aspects of the SUMO conjugation system. The yeast RAD60 carries three SLD domains each able to interact with different proteins in the SUMO-conjugation machinery [33]. RAD60-SLD2, although bearing surface charge different from SUMO has a similar face for interaction with the SUMO E2 and through this face may direct SUMOylation [33]. Similarly mouse Nip45 SLD2 interacts with UBC9 and inhibits the elongation of poly-SUMO chains [34]. The SLDs of UAF1, a partner protein to many de-ubiquitinating enzymes, is sufficiently similar to SUMO to interact with the SIMs in RAD51AP1 and in FANCI [35,36].

## 2. The fate of SUMOylated proteins

The catalogue of the mammalian cellular SUMO proteome, including targets in the DNA damage response (DDR), has expanded in recent years thanks to the efforts of several researchers using improved mass-spectrometry approaches to overcome the difficulties of mapping endogenous SUMO conjugation sites [16,22,37–44]. However for most individual proteins whether the modification has a functional role and what that role might be are not known. SUMO conjugation can block other PTMs at particular lysines, for example p53-K386 SUMOylation blocks its acetylation and subsequent DNA binding [45]. More often in targeted mutation experiments SUMO conjugation sites are found to be redundant and loss of a single SUMOylation site, with some exceptions (e.g. RanGAP [46], Sp3 [47], yeast PCNA (reviewed in [48])), has little impact on target-protein SUMOylation or target-protein function [26]. Nevertheless, SUMOylation can impact the function of a target protein through directing intra- or inter-molecular contacts via SIMs. Where SIM and SUMOylation site are found within a protein, SUMOylation can alter protein conformation. The most prominent example of this is thymine-DNA glycosylase, which recognizes mismatches in the process of base excision repair. SUMO conjugated at a single C-terminal lysine interacts with the nearby SIM promoting formation of a protruded α-helix within the catalytic domain, which is associated with reduced DNA binding [49–52].

When SUMOylation is restricted to a local area, such as at a DNA double-strand break (DSB), the SUMOylation ‘spray’ can promote protein group modification. This was first described in the yeast DDR, in which a SUMO conjugation wave brought about by the interaction of the E3 SUMO ligase Siz2 with DNA and Mre11 results in modification of protein groups promoting SUMO-SIM interactions between members of those groups [26,53]. Similarly in mammalian cells treatment with the alkylating agent methyl methanesulfonate (MMS) results in a network of SUMOylated proteins centred on PARP1 and histone acetyl transferase P300/CBP [11,41]. The ligase(s) that ‘spray’ this network are not yet identified.

Alternatively the interaction between SUMO-conjugated target and SIMs of a partner can result in the degradation of the target protein through the activity of SUMO targeting Ub (ubiquitin) ligases (STUbls), such as Arkadia/RNF111 or RNF4 (orthologue of *S. pombe* Rfp1 and Rfp2 and *Saccharomyces cerevisiae* Slx5/8 proteins) [54–56]. This class of protein interacts with the SUMO-modified proteins through tandem SIM motifs and directs the modification of the protein or the conjugated SUMO. STUbls [57,58], and possibly other ligases [59], may generate hybrid SUMO-Ub chains, indeed Ub is a significant target of SUMOylation [41]. Intriguingly the STUbl RNF4 is predominately monomeric and inactive in cells, but is activated by the presence of SUMO chains. On binding its tandem SIMs, SUMO chains promote RNF4 dimerization and ligase activity [60]. Thus RNF4 may ‘read’ highly SUMOylated proteins.

Functionally RNF4-mediated ubiquitination is often coupled to VCP/p97 (CDC48) activity. VCP is a multimeric ATPase that, with cofactors, is able to extract ubiquitinated proteins from membranes and protein complexes and direct them for proteasomal-mediated degradation (reviewed in relation to DSBs in this issue by Kristijan Ramadan and co-workers [61]). Deubiquitinating enzymes with the ability to remove Ub from SUMO-Ub chains include USP11 [57] and USP7 [62] although the degree to which these enzymes have a particular specificity for Ub conjugated to SUMO, rather than to another substrate, is not yet clear. In yeast a cofactor of the VCP homologue CDC48 called Ufd1 also binds SUMO [63]. This protein contributes to the displacement of SUMOylated proteins from DNA and in particular acts to restrain Rad51/Rad52 interactions [64]. Ufd1 is conserved in mammalian cells, and may perform a similar role [64], so that it is possible SUMOylation directs protein extraction without the need for a Ub conjugate intermediate.

**Figure 3.**
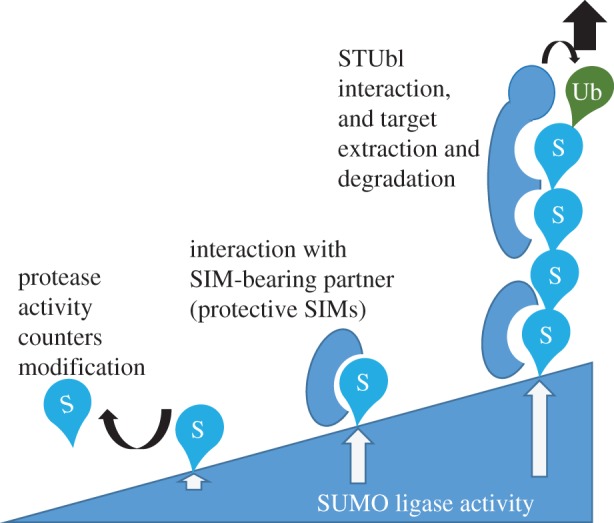
Proposed model of how SUMO conjugation and SIM-bearing proteins direct functional outcome for modified targets. The degree of SUMOylation regulates protein–target interactions.

While target-protein interaction with, and activity of, SUMO ligases versus SUMO proteases determine a protein's SUMO conjugation status, what then defines the fate of a SUMO modification is not yet obvious. Since the mediators of these fates chiefly interact with the same groove on SUMO isoforms, SIM-containing partners are likely to compete for SUMO on the modified protein, predicting that local partner proximity and relative concentrations are likely to regulate outcome. In addition some interactions may be a matter of degree, where high levels of mono-SUMOylation or polySUMOylation, not met with a competing, and ‘protective’, SIM, may favour interaction with STUbls resulting in protein extraction and loss (proposed model in figure 3)

## 3. The SUMO system in the DNA damage response

At micro-laser lines of DNA damage, prominent UBC9, PIAS1 and PIAS4 SUMO E3 ligase recruitment is observed together with weak SUMO E1 (SAE1) accumulation [65]. Moreover, SUMO1 and SUMO2/3 are detected in irradiation (IR)-induced foci (IRIF), laser-induced damage, and Lac arrays harbouring DSBs, and can be precipitated from damaged chromatin [65–70]. SUMO recruitment at damaged sites is dependent on the SUMO E3 ligases PIAS1/4 and on their DNA binding SAP domains [65]. In a parallel pathway the polycomb repressor complex 1 (PRC1) component and SUMO E3 ligase CBX4 are recruited through poly(ADP-ribose) polymerase (PARP) activity to sites of DNA damage [70]. Cells depleted for PIAS1 or PIAS4 have defects in the two main mechanisms of DSB repair, homologous recombination (HR) and non-homologous end-joining (NHEJ) and show sensitivity to and cisplatin [65,66], while CBX4 is also required for cellular resistance to IR [70]. Thus at least two pathways of SUMOylation are essential components of DSB repair.

It is possible that specific SUMO isoforms have particular roles at different portions of the response. Several reports have suggested that earlier arriving components of the DDR are modified by SUMO1 through PIAS4, and later arriving ones by SUMO2/3 through PIAS1 [31,65,71]. In one study SUMO2/3 preceded SUMO1 [67], whereas others observed simultaneous accumulation [66,68], and CBX4 loss reduces both SUMO1 and SUMO2/3 accumulations at DSBs [70].

The STUbl RNF4 plays multiple roles in DSB repair. Its recruitment to sites of DSBs is dependent on its SIM motifs, on SUMO2/3 and on PIAS1/4 SUMO ligases [68,72], and it can be detected at laser lines within seconds of exposure, persisting for several hours [68,69,72,73]. The presence of several DNA repair factors improves RNF4 localization to breaks, including SUMO targets MDC1, RNF8, 53BP1 and BRCA1 [68,72], whereas RNF4 depletion results in the persistence of SUMO isoforms at laser line-induced damage [72]. RNF4, and its yeast orthologues, interact directly with nucleosomes and DNA and have a role in promoting ubiquitination of H3 and telomere repair, but whether this activity also relates to a role in global DSB repair is not known [74,75]. It is possible RNF4 is primed for chromatin substrates in this way.

Intriguingly cycles of SUMO deconjugation appear critical to the DDR. Expression of SUMO mutants that cannot be deconjugated are disruptive to DSB repair [76]. Moreover, knockdown of individual SENP enzymes, with the exception of SENP3, each results in specific alterations in HR/NHEJ efficiencies measured after a DSB of integrated reporters [76]. Some enzymes are likely to have dramatic impacts on SUMO availability, either through directing SUMO maturation or by allowing the release of free SUMO from conjugates. For example loss of the Ulp2-related protease SENP6 dramatically increases the amount of high molecular weight SUMO2/3 conjugates and enlarges SUMO in promyelocytic leukaemia protein (PML) nuclear bodies, suggesting it is responsible for a large proportion of cellular SUMO2/3 editing [77]. The requirement for SENP6 in assays of NHEJ and HR repair is rescued by supplying exogenous SUMO isoforms, suggesting SUMO supply through SENP6 activity is required in the DDR [76]. Other enzymes are likely to restrain SUMOylation of a restricted subset of substrates. For example the requirement in HR repair for the Ulp2-related protease SENP7 cannot be rescued by increased SUMO supply and instead SENP7 has a role in the chromatin relaxation required to allow DNA repair [76]. Non-redundancy and clear differences in localization and isoform specificity suggest further investigation will identify specialist functions for SUMO proteases in DNA repair.

## 4. SUMO repression of chromatin at DSBs

**Figure 4.**
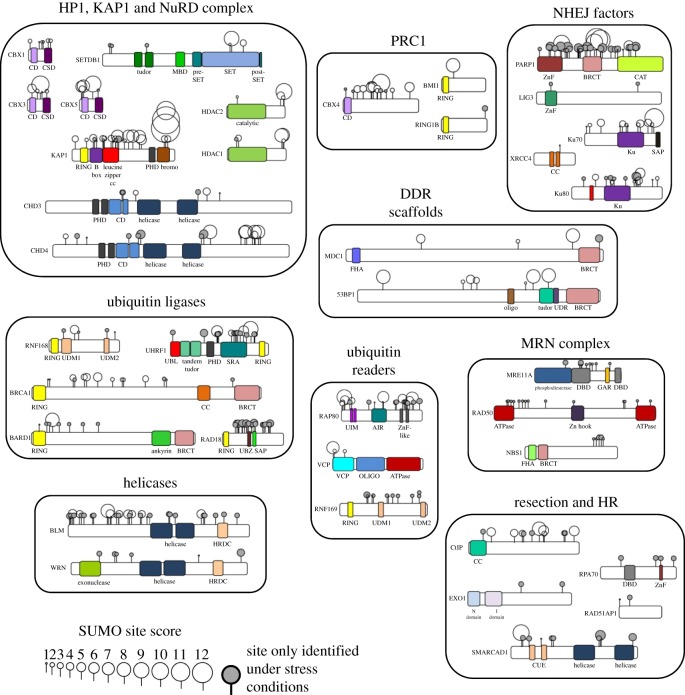
SUMOylated factors involved in DSB repair response. Proteins are grouped according to their enzyme class or functional complex. SUMO sites are from Hendriks & Vertegaal 2016 [11], which compiled every SUMO site-mapping proteomic analysis published up to 2016. Sites are given a score depending on their occurrence within different datasets, such that sites in multiple datasets are represented by larger circles. The functional dominance of these sites has not been determined in most cases. Grey circles denote sites only found in cells that have been treated with stress; for details see reference [11]. The majority of studies were performed using SUMO2, and individual SUMO isoforms are not shown. Proteins are shown to scale with only the longest isoform being used. Domain locations are taken from UniProt. Abbreviations; BRCT (BRCA1 C-terminus), CC (coiled coil), CD (chromodomain), CSD (chromo-shadow domain), CUE (Cue ubiquitin binding), DBD (DNA binding domain), FHA (forkhead-associated), GAR (Gly/Arg-rich), HRDC (helicase and RNaseD C-terminal), MBD (methyl binding domain), NET (N-terminal extra-terminal domain), OLIGO (oligomerization domain), PHD (plant homeodomain), RING (really interesting new gene), SAP (SAF-A/B, Acinus and PIAS), SRA (SET- and RING-associated domain), SPRY (SP1a and ryanodine receptor), SQ/TQ (region rich in SQ/TQ phosphorylation sites), UBL (ubiquitin-like), UDM (ubiquitin-dependent recruitment module), UBZ (ubiquitin binding zinc finger), UDR (ubiquitin-dependent recruitment), ZnF (zinc finger).

Chromatin context is critical for DNA repair outcome and chromatin-related ontologies are consistently highly ranked in SUMO proteomes, with many of the most highly SUMOylated proteins having roles in chromatin architecture. SUMO modification contributes to transcriptional repression in several contexts [78–80], and SUMO is central to the maintenance of heterochromatin [81,82]. Moreover, SUMO is found coupled to multiple repressor complexes, in which SIM-bearing proteins are also enriched [83]. SUMOylation induced by DNA damage can influence patterns of gene expression through specific factors such as HIC1 (*H*ypermethylated *I*n *C*ancer 1) [84–86]. Further, SUMO is part of the repressive chromatin environment associated with the rapid and transient recruitment of complexes to chromatin around DSBs. These include NuRD and HDACs, the Suv39H1/KAP1/HP1 complex, lysine demethylases and polycomb repressor complexes (figure 4). Together these induce histone deacetylation and increased H3K9me2/3 and increase nucleosome packing. This initial repressive state is thought to prevent inappropriate chromatin movement, to maintain the relationship between DNA ends, and to contribute to local transcriptional silencing (reviewed in [87]). However for DNA repair to proceed an open chromatin structure is needed and overcoming SUMO-mediated repression is part of the transition to a permissive chromatin environment [88]. Examples of SUMO in chromatin repression and release are described below.

SUMOylation of the polycomb repressor complex 1 (PRC1) contributes to its recruitment to sites of DNA damage [70]. SUMOylation of the PRC1 Ub ligase component BMI1 at K88 in response to IR requires the PRC1 component and SUMO ligase CBX4. BMI1, together with its partner RING1A/B, functions as a Ub ligase, and ubiquitinate H2A at K119 both at promoters and at sites of damage [89–91]. At damage sites it contributes to local transcriptional repression [92–94] and influences DDR Ub-signalling [90,95]. PRC1 recruitment had been thought to be hierarchical though the methylation of chromatin via the PRC2 complex; however recent findings indicate that PRC2 can be recruited by PRC1 modification of H2A-K119-Ub [96,97]. Thus an initial recruitment of PRC1 promoted by PARP activity and SUMO [70] could seed further spreading of the complex at sites adjacent to DSBs.

Methylation on H3K4, H3K36 and H3K79 is generally associated with active gene expression [98] with H3K4me3/2 residues marking the transcriptional start sites of actively transcribed genes. Intriguingly the H3K4m2/3 demethylases JARID1B and JARID1C are both SUMOylated in cells exposed to MMS but with differing consequences. SUMOylation of chromatin localized JARID1B is associated with its RNF4-mediated proteasomal degradation. In contrast SUMOylation of the nucleoplasmic JARID1C coincides with its chromatin recruitment and with global demethylation of H2K4me3 and transcriptional downregulation. In addition the H3K9me3 mark, increased on DNA damage [99] and associated with transcriptional repression, may be associated with SUMOylation of MBD1 and SETDB1 [41].

One of the most abundantly SUMOylated proteins in human cells is the transcriptional repressor and heterochromatin nucleator protein KAP1/TRIM28 ([11] and figure 4). KAP1 possesses auto-SUMO ligase activity within its PHD domain and the majority of its SUMOylated lysines are found in the C-terminal bromodomain (figure 4) [100]. SUMOylation of KAP1 promotes interaction with the SIMs of the NuRD subunit CHD3 and the methyltransferase SETDB1 [100]. This recruitment promotes histone deacetylation and chromatin remodelling via the HDACs and CHD helicases contained within the NuRD complex and H3K9 trimethylation by SETDB1 which in turn promote gene silencing and chromatin compaction. KAP1 interacts with HP1α, which binds to trimethylated H3K9 [101–103] so that the propagation of the H3K9me3 along chromatin attracts additional HP1α proteins which then recruit additional KAP1 molecules, allowing spreading of heterochromatin, or a heterochromatin-like state [104].

KAP1 localizes to sites of damage [105], and initially spreads a repressive environment along tens of kilobases [106]. Critically, the repressive influence of the SUMOylated protein in this context is then attenuated by the DDR. This occurs through activation of the Tip60 acetylase and activation of ATM. ATM activity evicts the H3K9me2/3 methylase SUV39H1, and releases the repressive HP1/KAP1 complex [106] via phosphorylation of chromatin- associated KAP1 at Ser^824^ adjacent to its bromodomain [107]. This phosphorylation event also disrupts the SUMO-SIM interaction with CHD3 of the NuRD complex. The subsequent release of CHD3, like depletion of KAP1 itself [108], promotes chromatin relaxation and allows DNA repair [107]. Heterochromatin contains more repressive complexes and exhibits slower DSB repair (reviewed in [109]) and the impact of ATM-mediated KAP1 phosphorylation on repair kinetics is most dramatically observed in these more condensed regions [107,110,111].

Such a finely balanced mechanism suggests perturbation of SUMOylation at chromatin might impact chromatin state and subsequent DNA repair proficiency. Indeed cells depleted of the chromatin-associated SUMO protease SENP7 fail to relax chromatin following DNA damage [76]. Consequently, this enzyme is required for resistance to DNA damaging agents and to promote HR repair [76]. Like KAP1, SENP7 also interacts with HP1α [76,112] and is enriched in heterochromatin [112]. It acts to deSUMOylate KAP1 [76] and HP1α [112,113]. Chromatin association of the NuRD component CHD3 is increased in cells without SENP7 and importantly the requirement for SENP7 in promoting chromatin relaxation, HR-repair and resistance to IR is diminished if CHD3 is depleted [76]. Thus it appears that SENP7 is needed to restrain KAP1-CHD3 interaction by cleaving KAP1-SUMO conjugates. Accessibility in euchromatin (transcribed genes) is severely reduced without SENP7, but DNA damage markers also persist for longer in regions of heterochromatin [76], suggesting it too is critical to DSB repair in heterochromatin. KAP1-SUMO is also counteracted by the RNF4-VCP pathway. Both RNF4 and VCP/p97 interact with pS824-KAP1-SUMO and RNF4 promotes the degradation of SUMOylated KAP1 [114,115], providing a further mechanism of chromatin derepression.

Thus the promotion of repressive chromatin by SUMO interactions appears initially co-opted in the DDR, to promote the transient accumulation of repressive complexes and to mediate DSB-associated transcriptional repression. However it must then also be overcome to permit adequate repair. Perhaps consistent with this notion is the finding that SUMO conjugation sites in many proteins involved in chromatin organization and modification are lost following MMS treatment [41]. Thus widespread deSUMOylation or SUMO-mediated degradation of chromatin-associated targets appears a significant, and still largely unexplored, cellular response to DNA damage.

## 5. SUMO in non-homologous end-joining

Throughout the cell cycle the most prominent form of DSB repair is the error-prone pathway of non-homologous end-joining (NHEJ). This process is reviewed extensively elsewhere [116], but in simplified form the Ku70/80 heterodimer initiates end binding, encircling the DNA, and recruits the DNA-PK catalytic subunit. Complex ends are then trimmed to blunt ends by proteins such as Artemis or PNKP and the XRCC4/XLF/PAXX complex stabilizes DNA ligase IV which joins the DNA ends together. Several NHEJ factors are SUMO modified in mammalian cells (figure 4) but any influence on function is understood for just a few factors. XRCC4 SUMOylation is linked to its nuclear localization [117]. Cells expressing a form of XRCC4 with an arginine at the K210 SUMOylation site show a cytoplasmic localization coupled with sensitivity to IR and poor V(D)J recombination. Importantly fusing SUMO to the C-terminus restores normal localization of XRCC4 and radio-resistance [117]. In yeast, SUMOylation enhances the DNA association of Ku70 [118] and in mammalian cells increased expression of SUMO conjugation components causes Ku70 stabilization, although this effect may be indirect [119]. Nevertheless, a role for SUMO interactions in mammalian NHEJ is suspected as expression of SIM-peptides can block NHEJ and increase cellular radio-sensitivity [120]. These peptides immunoprecipitate SUMO-Ku70, suggesting an as yet unidentified interaction with modified Ku70 may be significant in the repair process [120]. Perhaps consistent with these observations is the finding that loss of RNF4, which bears tandem SIM motifs, reduces NHEJ repair outcomes through an as yet unknown pathway [72,115]. Ku70/Ku80 encircle DNA ends [121], where they prevent extensive resection, and promote DNA-PKcs (DNA-dependent protein kinase, catalytic subunit) recruitment. However after DNA ligation Ku70/80 rings remain locked on the DNA and are deleterious unless removed [122]. RNF8, RNF138 and SCF ubiquitin ligases have been implicated in the disengagement of Ku80 [123–125] and VCP/p97 and its ubiquitin binding receptors have been shown to be required for extraction of both Ku70 and Ku80 from damaged DNA [126]. As each Ku subunit independently encircles the DNA [121], an attractive, but as yet untested, model for removal of Ku70 is its SUMO-mediated Ub targeting and subsequent extraction.

## 6. SUMO in DNA damage response signalling

DNA double-stranded breaks (DSBs) are also recognized by a signalling complex composed of NBS1, RAD50 and MRE11A (MRN). MRN helps to recruit the kinase ATM which phosphorylates multiple components of the DSB pathway, including the histone variant H2AX at Ser^139^ [127]. MDC1 is recruited to phosphorylated H2AX through its BRCT domain and is critical to subsequent DSB signalling [128–131]. It begins a Ub signalling cascade involving the E3 Ub ligases RNF8/RNF168, Ub conjugating enzyme, UBE2N/UbcH13, and two factors that promote RNF8 interactions, HERC2 and JMJD1C (reviewed elsewhere [132]). SUMO-dependent recruitment of the PRC1 complex brings the BMI1 : RING1A/B Ub ligase [70] which then contributes to activation of the Ub signalling pathway [90,92,95,133] (although its recruitment has been disputed [134]). This Ub cascade results in the recruitment of the BRCA1-A complex and 53BP1.

MDC1, HERC2 and RNF168 are SUMO modified by PIAS4 following DNA damage [31,40,43,68,71]. The demethylase JMJD1C which binds to RNF8 and MDC1 [135], the Ub E2 enzyme UBE2N/UbcH13 which cooperates with RNF8 and RNF168, and RNF8 itself are also SUMOylated at multiple sites, although it is not known which ligases are responsible, nor what function their SUMOylation may have [15,31]. These proteins could be seen as a protein group, co-located in time and space and therefore an example of the SUMO spray model [53]. However, in a departure from that model, SUMOylation of at least some individual components has distinct functional outcomes.

MDC1 SUMOylation drives RNF4 interaction after exposure to IR [68,71]. A single lysine, K1840, appears to be the major site of MDC1 SUMOylation [71] and in cells without RNF4 or PIAS4, or expressing K1840R-MDC1, the protein shows slowed clearance from sites of damage [71,72]. Moreover, K1840R-MDC1 fails to rescue radio-sensitivity of MDC1-deficient cells. The RNF4-mediated ubiquitination of SUMOylated MDC1 is antagonized by the DUB ATXN3 which is recruited to DSBs in a SUMO-dependent manner. ATXN3 therefore acts as a brake on MDC1 turnover at DSB which is essential for proper downstream signalling and HR/NHEJ repair efficiencies [73]. The consequences of prolonged association of MDC1 at damage sites is reduced HR repair, potentially through an increased 53BP1 association [71]. In contrast SUMOylation of HERC2 relates to the promotion of an inter-molecular structural interaction, in which the ZZ-type zinc finger in HERC2 is suggested to mediate interaction with SUMO-modified HERC2, resulting in either a conformational change or novel interaction that regulates its ability to interact with RNF8 [31]. In yet another functional outcome the SUMO E3 ligase PIAS4 supports RNF168 protein stability and promotes its transcription [31].

The RNF8/RNF168 Ub cascade results in the recruitment of the BRCA1-A complex to sites of DSBs through the K63-Ub linkage and SUMO sensor RAP80, a component of the BRCA1-A complex [136–142]. In addition to a dual Ub-interacting motif (UIM) [139,143] RAP80 carries a SUMO-interacting motif (SIM). These bind Ub-SUMO hybrid chains with high affinity [143]. Both the SIM and UIM domains are required for efficient recruitment of RAP80 to DSBs immediately after damage and confer cellular resistance to ionizing radiation [67]. The K63-Ub-SUMO bound by RAP80 is reported to be provided by RNF4 [58], which is capable of generating K63-linked Ub chains [144]. Further work is needed to establish the degree to which SUMO binding mediates the interaction of RAP80 with the K63-Ub chains generated by RNF8/168, which are also required for RAP80 recruitment [145], and whether SUMO-Ub hybrids occur on any specific substrate(s).

BRCA1 and 53BP1 recruited to sites of DSBs are cell cycle specific gate-keepers to the critical HR step of DNA resection, where the 5′ ends of the DSB are subject to nuclease processing. 53BP1 acts to restrain resection in G1, and BRCA1 acts to promote it in late S and G2 [146–149]. BRCA1 is SUMOylated in response to IR and other DNA damaging agents [41,65,66,68] through PIAS1/4 SUMO ligases at several sites including at its N-terminus (figure 4). SUMOylation is associated with increased BRCA1 : BARD1 Ub ligase activity *in vitro* [66] although whether this increased activity relates to SIMs in Ub conjugating enzymes, or is structurally related to a similar potentiation of ligase activity induced by auto-ubiquitination [1,150], is not clear. The functional role of the Ub ligase activity of BRCA1 in DDR has for many years been controversial, but recent reports suggests it promotes Ub modification of the extreme C-terminus of H2A [151], which in turn encourages the recruitment and activation of the remodeller SMARCAD1 resulting in increased long-range DNA resection [95,152]. Whether SUMOylation of BRCA1 : BARD1 also potentiates resection remains to be seen and the relationship of BRCA1 SUMOylation with the different BRCA1 complexes, of which only one is expected to promote resection, is currently unknown. SUMOylated BRCA1 is a substrate for RNF4 [69], suggesting its clearance also requires SUMOylation.

53BP1 is a reproducible substrate of SUMO modification on DNA damage [65,68] with several sites mapped (figure 4). Currently there are no reports of mutation of the sites, or SUMO fusion, or any impact of RNF4 or VCP/p97 directly on 53BP1. Loss of PIAS4 reduces 53BP1 localization [65], but this may be an indirect effect through the ligase's impact on RNF168 [153]. 53BP1 acts to promote NHEJ and inhibit resection of DNA ends through its effector proteins, PTIP, RIF1, Artemis and Rev7. These have not yet been identified as SUMOylated and the role, if any, of 53BP1 SUMOylation awaits investigation.

## 7. SUMO in resection and recombination

In late S-phase and G2 stages of the cell cycle error-free DNA repair is possible using the sister chromatid as a template for HR repair. The 5′ resection of the double-stranded DNA to produce 3′ overhangs, required for HR, employs the enzymatic activities of Mre11-CtIP, EXO1 and BLM/DNA2 (reviewed extensively elsewhere [154]). The single-stranded DNA (ssDNA) produced is first bound by RPA, and then exchanged by the action of BRCA2 for RAD51. Recent data suggest an emerging role for SUMO in this process, as most proteins with a direct role in resection are SUMO modified. Components of the MRN complex, including MRE11, are SUMOylated following infection with adenovirus 5 (Ad5), which triggers a DSB response as the ends of the viral genome mimic host cell DSBs [155,156] and multiple SUMO sites have been identified on the MRN components from SUMO site-mapping screens (figure 4). BLM is SUMO modified in response to IR and replication stress [69,157] and after replication fork collapse SUMO-modified BLM is implicated in promoting RAD51 foci formation [157,158]. EXO1 is SUMOylated by PIAS4 which reduces its stability, whereas loss of the SUMOylation sites of EXO1 improves the protein's stability [159]. In addition the ssDNA binding protein subunit RPA70/RPA1 itself bears a critical SUMO-modification site which is required for subsequent RAD51 accumulation [160]. Moreover, both RPA1 and EXO1 are bound by the SUMO protease SENP6 which is reported to promote their hypoSUMOylation [159,160].

These observations suggest that the resection process is SUMO rich, and might be ‘read’ by specific mediators. Intriguingly both the scaffold protein for many structure-specific endonucleases, SLX4, and RAD51 carry SIM motifs critical to their function [161,162]. SLX4 localization to laser-induced damage requires its SIMs and the SUMO pathway [162]. The SLX SIMs are required for normal cell resistance to camptothecin, suggesting a role related to DSBs associated with replication-associated DNA damage. SUMO binding increases SLX4 interaction with MRN and RPA [162] and although SLX4 binds to SUMOylated forms of these proteins recruitment is also thought to be promoted by other SUMO targets, to account for its SUMO-dependent recruitment.

Similarly SUMO concentration may promote the ability of RAD51 to ‘read’ resection. A direct interaction between RAD51 and SUMO was first established by yeast two hybrid interaction [163]. RAD51 contains a conserved C-terminal SIM (VAVV 261–264) and mutation of this sequence abrogates RAD51 accumulation at laser lines and reduces HR-mediated repair [164]. The interaction partner(s) of this SIM is not known but candidates in the resection processes are appealing.

The potential for SUMO-targeting Ub ligases to regulate aspects of resection and recombination is therefore significant and indeed several studies agree that loss of the STUbl RNF4 is associated with reduced RAD51 foci formation and poor HR outcome [68,71,72]. However while two studies suggest the defect in cells without RNF4 is at the level of reduced resection [68,71], perhaps due to a failure to clear MDC1 and in turn 53BP1 [71], another suggests RNF4-mediated turnover of SUMO-RPA1 determines RPA residency at already resected DNA, resulting in reduced RAD51 loading [72]. Taking resection and recombination together it would appear that SUMO plays several, sometimes counteracting, roles, promoting protein stability and interaction but also clearance, depending on the substrate. It is perhaps not surprising that different studies locate different DDR pressure points that the RNF4 ‘wrecking ball’ is required to transit. Indeed it seems likely that there are yet further roles for RNF4 in this pathway to be discovered.

## 8. Conclusion and questions

Insight into how SUMO modification coordinates the fundamental process of DNA double-strand break (DSB) repair is emerging. Indeed this may contribute to further understanding of the established role SUMOylation has in ageing and senescence [165], and neurological disorders [166]. The role of SUMO in transcriptional repression and maintenance of heterochromatin appears co-opted in the acute response to damage but is then overcome to open chromatin structure. Given the differing chromatin environments that DSBs arise in, the possibility that SUMO influences different repair outcomes in differing contexts appears likely.

The catalogue of SUMOylated proteins in the DDR is expanding [11] but a striking conclusion of surveying examples of SUMO modification in the mammalian DDR is the varied ways in which SUMO impacts protein function, even when an apparent protein group is modified. The DDR uses all variants of SUMO target fate to drive interactions and transitions that permit the response to proceed. Our current overview of SUMO function in the DDR points to several points of precision and instances of opposing functional outcome of SUMO modification of similarly located proteins. Thus an indiscriminate SUMO ‘spray’ and subsequent protein group modification directing the accumulation of SUMO : SIM-associated complexes prevalent in the yeast DDR [26] would appear to be a less dominant mechanism of SUMO function in the mammalian response.

The steady-state of target-protein SUMO modification is a consequence of conjugation/deconjugation and target-protein stability, and thus the majority of our cataloguing of SUMOylated targets perhaps represents sites less amenable to isopeptidase or STUbL access. While work developing our understanding of SUMO conjugation is growing, an understudied area is SUMO protease regulation. The constitutively high activity of these enzymes means that regulation at the level of modification removal may be as significant to target proteins and protein groups as the conjugation itself. Since SUMO proteases are potential targets for small molecules [167–169], with potential utility in several human disease states, there is a need to further establish their roles. Future SUMO site-mapping experiments, identifying SUMO targets in cells with removal or inhibition of specific SUMO proteases and STUbls, will greatly improve our understanding of the dynamics of SUMOylation in different sub-nuclearcompartments and after particular stimuli, such as DSBs.

SUMOylation exhibits considerable cross-talk between PTM pathways, as described herein with Ub modification, but also with phosphorylation [15,16] and thus investigation of SUMO and SUMO targets is necessary to gain an integrated view of how DSB repair is orchestrated. This point is conspicuous when thinking about the dense signalling environment of chromatin. For example H2AX is also SUMOylated by PIAS4 in response to various DNA damaging agents [170] with major conjugation sites at K128 and K135 [15,37]. SUMO is a bulky adduct relative to histones and nucleosome structure with potential to impact packing or affect interactions with nearby phosphorylated S139 or Ub-modified K119/120.

Finally, as SUMO site mapping reaches saturation a key goal for the future is to establish the mechanisms and circuits of SUMOylation in the DDR and their integration into our wider understanding of the response. By these means, how SUMO dynamics contribute to maintaining a stable genome, and how these circuits might be exploited, will become clear.

## Data accessibility

This article has no additional data.
